# Soil microbial diversity under different types of interference in birch secondary forest in the Greater Khingan Mountains in China

**DOI:** 10.3389/fmicb.2023.1267746

**Published:** 2023-10-23

**Authors:** Kaitao Zhai, Yongchun Hua, Jingwen Liang, Jing Li, Zirui Wang, Lei Liu, Minglong Gao, Rula Sa, Mingmin Zhao

**Affiliations:** ^1^College of Forestry, Inner Mongolia Agricultural University, Hohhot, China; ^2^College of Horticulture and Plant Protection, Inner Mongolia Agricultural University, Hohhot, China

**Keywords:** Greater Khingan Mountains, birch secondary forest, bacterial diversity, fungi diversity, forest ecological cycle

## Abstract

**Introduction:**

Soil microorganisms are an important component of soil ecosystems with an indispensable role in forest ecosystems. We analyzed the soil microbial diversity in birch secondary forest formed by natural restoration or artificial reconstruction after interference by burning, clear cutting, and gradient cutting, and the *Betula platyphylla Suk* undisturbed forest in the Greater Khingan Mountains in China.

**Methods:**

Illumina high-throughput sequencing technology was used to analyze the characteristics of the soil microbial community during the restoration process of birch secondary forest caused by the different types of interference. The relationships between bacteria and fungi were analyzed. The gene functions of the soil bacterial community and the ecological functions of soil fungi were predicted using PICRUSt and FunGuild, respectively.

**Results:**

At the phylum level, the species and quantity of bacteria were more abundant than that of fungi. At the genus level, no obvious differences in the abundance of bacteria were observed; there were obvious differences in the abundance of fungi. Among the eight sample plots, the artificial larch forest belt had the highest bacterial and fungal alpha diversity, which was slightly higher than undisturbed forest, while the other sample plots were significantly lower. Gradual cutting pure birch forest bacteria and fungi had the highest beta diversity, and artificial larch forest belt bacteria and heavy burn sample plot fungi had the lowest beta diversity. Samples from the cutting and burning sample plots were significantly different from the undisturbed forest at the phylum level of Acidobacteriae, Acidimicrobiia, Mortierellomycetes and Sordariomycetes. We found statistical differences in biomarkers between bacterial and fungal communities in undisturbed forest and artificial larch forest belt and burn sample plots. PICRUSt prediction and FunGuild prediction showed that soil bacterial and fungal communities were rich in gene and ecological functions, respectively. In the microbial network, the stability or anti-interference performance of the fungal community was higher than that of bacteria.

**Conclusion:**

Our data reveal the characteristics of the soil microbial community during the restoration process of *Betula platyphylla Suk* secondary forest under different types of disturbance, which is of great significance for understanding the role of soil microorganisms in the forest ecological cycle.

## 1. Introduction

Soil microorganisms are an important component of soil ecosystems and play an indispensable role in the material circulation and energy flow of forest ecosystems (Chongbang et al., 2003). Soil microbial diversity can be defined as the richness of microbial life, which is usually reflected in the interrelationship between changes in soil biota and biochemical processes (Yang H. J. et al., 2005). The taxonomic composition and diversity of soil microorganisms are important biological indicators for reflecting the formation and evolution of soil fertility (Diacono and Montemurro, 2010), evaluating the relationship between biomes and restoration functions in degraded ecosystems (Harris, 2003), and warning of ecosystem changes (Bell et al., 2005).

Among the soil microorganisms, bacteria are the most abundant and widely distributed group (Chen et al., 2011; Delmont et al., 2012; Fierer et al., 2012). Soil bacteria participate in the transformation of the nutrient elements in soil, actively promoting the formation and decomposition of organic matter. Bacteria also play an irreplaceable role in the improvement of the ecological environment (Kennedy, 1999; Nacke et al., 2011). It has been reported that the higher the diversity of soil bacteria, the stronger the sustainable utilization and pressure resistance of soil (Zhou et al., 2002). Most fungi in forest soils are more sensitive than bacteria in the face of changes in the physical and chemical properties of the soil and ground vegetation, and have a greater impact on the material circulation in the forest (Urbanová et al., 2015; Luo et al., 2017). Fungi mainly participate in the decomposition of complex high carbon and nitrogen organic matter (Blagodatskaya and Anderson, 1998) with a slow decomposition rate (Fan and Hao, 2003) and small quantity (Yang T. et al., 2005), which requires relatively stringent environmental conditions. The species and quantity of fungi are important indicators for evaluating an ecosystem (Diacono and Montemurro, 2010). Studies have shown that bacteria are more suited to broad-leaved forests with sufficient water and fertilizer and easy decomposition, whereas fungi are more suited to coniferous forests with poor soil and difficult decomposition (Albiach et al., 2000; DeGrood et al., 2005).

However, various types of disturbance frequently exist in nature, and different types of disturbance have particular impacts on the forest ecosystem. Excessive disturbance will cause serious degradation of the forest soil and further affect the sustainable development of the forest ecosystem (Atkins et al., 2020). Among them, cutting and fire disturbances are the main causes of forest soil degradation (Ghazoul et al., 2015; Silvério et al., 2019). In forest ecology, fire, as a major disturbing factor, can significantly change the structure and function of the forest ecosystem (González-Pérez et al., 2004; Shakesby et al., 2007; Keesstra et al., 2017). Fire disturbance can directly kill soil microorganisms through high temperature (Sadeghifar et al., 2020), or indirectly affect the structure and function of the soil microbial community through changes in the physical and chemical properties of the above-ground vegetation and soil (Certini, 2005; Muñoz-Rojas et al., 2016; Alcañiz et al., 2018; Sánchez-García et al., 2020). In the process of forest burning, the soil surface temperature can reach to 50–1,500°C (Sadeghifar et al., 2020), during which the bacteria and fungi in the soil are quickly killed at 50–150°C (Santín and Doerr, 2016). After fire disturbance, soil bacteria recover first, with a shorter recovery time than that of fungi (Tao, 2013). The diversity of soil bacteria gradually recovered with the increase of time after fire, and the effect of fire on soil fungi was more significant than that of soil bacteria. Soil fungal community structure showed significant changes both in the short term and the long term after fire (Sun et al., 2016; Duhamel et al., 2019; Wang et al., 2022). In addition, it has been found that soil fungal richness and diversity decreased with the increase of fire severity (Day et al., 2019).

The impact of logging on the forest ecosystem is complex. After a forest is disturbed by logging, sunlight directly hits the ground, causing a large loss of soil nutrients and changes in the soil temperature and humidity, forcing the soil function to decline (Sheng, 2014). In addition, logging also reduces the input of soil organic matter by ground vegetation, thus affecting the structure and function of the microbial community (Ahn et al., 2012). Many studies have shown that soil microorganisms are suited to living in areas with slight sunlight penetration (Adekunle et al., 2005). In lightly logged forests, the diversity of bacteria and fungi are higher, while heavy logging results in low fungal diversity (Olusola and Adeduntan, 2016). In addition, mild logging disturbance can help protect the stability of the soil fungal community, while severe logging disturbance can significantly reduce the number of soil fungi (Chatterjee et al., 2008; Tomao et al., 2020). However, the species and quantity of soil bacteria in unlogged forests are higher than those in logged forests (Fan, 2016).

*Betula platyphylla Suk* is the main pioneer tree species in the areas with disturbance caused by fire and logging in the Greater Khingan Mountains (Gao, 2020). The secondary forest of *Betula platyphylla* occupies 39.2% of the forest area. It plays an important role in the soil and water conservation, climate regulation, and maintenance of the ecological balance (Fan and Yan, 2013), which is forming an indispensable ecological barrier in Inner Mongolia in China (Zhu et al., 2013). It also has important economic value. Secondary forest refers to stands formed after natural or artificially induced regeneration due to major changes in the basic structure and function of the primary forest (caused by destructive human or abnormal natural disturbances) (Zhu and Liu, 2007). Soil microbial communities play an important role in regulating both organic matter degradation and nutrient cycling in forests (Treseder et al., 2004), and maintaining the structure and function of forest ecosystems in cold regions (Jiang et al., 2021). The research on soil microorganisms of *Betulae alba* secondary forest formed under different types of disturbance is of great significance to reflect forest stability and stress resistance and to evaluate forest health, and provide a scientific basis for achieving sustainable forest management. In this study, to explore the characteristics of the soil microbial community during the restoration process of birch secondary forest caused by different types of interference, Illumina high-throughput sequencing technology was used to analyze the soil microbial diversity in birch secondary forest formed by natural restoration or artificial reconstruction after the three interference modes of burning, clear cutting and gradient cutting, and the undisturbed *Betula platyphylla Suk* forest in the Greater Khingan Mountains in China. The relationships between bacteria and fungi were also analyzed.

## 2. Materials and methods

### 2.1. Location of the study areas

The study area was located in the northern forest area of the Greater Khingan Mountains in Inner Mongolia (120°12′ –122°55′E, 50°20′ –52°30′N), and the sample plots were set in Chaozha Forest Farm, Murui Forest Farm, and Upper Yanggeqi Forest Farm of Genhe Forestry Bureau. The forest area is mainly flat mountain at altitude 700–1,530 m, with a cold temperate humid monsoon climate, which has an average annual temperature of −5.3°C and average annual precipitation of 500 mm. The soil type is mainly brown coniferous forest soil, containing sand and gravel, and the soil is acidic. The study area is rich in species, mainly in the cold temperate coniferous forest with *Larix gmelinii* as the established species, accompanied by many *Betula platyphylla* secondary forests. The main undergrowth plants are *Vaccinium vitis-idaea* L., *Ledum palustre* L., *Rhododendron simsii* Planch., *Carex tristachya* Thunb., *Deyeuxia purpurea* (Trin.) Kunth and *Melica virgata* Turcz. ex Trin.

### 2.2. Design of different interference types

The design of the test sample plots with the different interference types are shown in Table 1.

**Table 1:** Design of test plots with different interference types.

Interference mode	Sample plots	Elevation (m)	Tree species	Canopy density	Total forest storage (m^3^/ha)	Slope	Aspect
Overcutting disturbs the plot	Gradual cutting sample S1	969	Pure *betula platyphylla*	0.76	9.8995	2°	Southwest slope
	Gradual cutting sample S2	973	9 *Betula platyphylla* 1 *Larix gmelinii*	0.642	11.9829	2°	Western slope
	Gradual cutting and replenishing sample plot S3	940	8 *Betula platyphylla* 2 *Larix gmelinii*	0.64	5.99095	2°	Southwest slope
	Gradual cutting and replenishing sample plot S4	910	6 *Larix gmelinii* 4 *Betula platyphylla*	0.6	9.1455	4.5°	Southwest slope
Artificial modification interferes with the sample	Modified sample plot ML by 50 m artificial larch forest belt	710	9 *Larix gmelinii* 1 *Betula platyphylla*	0.6	16.1702	8°	West slope
	Unchanged, pure natural forest MB	705	9 *Betula platyphylla* 1 Populus davidiana	0.6	11.3353	8°	West slope
Fire disturbance	Heavy burn Sample plot H1	940	9 *Betula platyphylla* 1 *Larix gmelinii*	0.3	0.4258	4°	West slope
	Moderate fire Sample plot H2	940	Pure *betula platyphylla*	0.44	1.5504	2.3°	West slope

### 2.3. Soil sample collection

In the study area, a total of eight sample plots were set up in the secondary birch forest formed by fire, clear cutting, gradual cutting and no disturbances, and three soil plots were set up evenly, diagonally in the sample plots. The surface humus layer was removed, and samples were taken from 0–20 cm of each soil square. The soil samples were sorted into 10 mL centrifuge tubes through a 2-mm sieve and numbered. The soil samples were stored at −20°C for transport to the laboratory for DNA extraction. A total of 24 soil samples were collected from the eight sample plots.

### 2.4. Analysis of soil microbial by high-throughput sequencing

The CTAB (cetyltrimethylammonium bromide) method was used to extract the total DNA of the microbiome samples from the various sample plots (Siregar et al., 2021). The extracted DNA quality was determined using agarose gel electrophoresis, and the DNA was quantified using a UV spectrophotometer. The thermal cycling conditions for bacteria and fungi began with an initial denaturation at 98°C for 30 s. For bacteria this was followed by 35 cycles at 98°C for 10 s, 55°C for 30 s, and 72°C for 45 s. For fungi this was 32 cycles at 98°C for 10 s, 54°C for 30 s, and 72°C for 45 s. The final extension for both was 10 min at 72°C. For bacteria, the primers 515F (5′-GTGYCAGCMGCCGCGGTAA-3′) and 806R (5′-GGACTACHVGGGTWTCTAAT-3′) were used for PCR amplification of the 16S rRNA variable region (V4). For fungi, ITS5-1737F (5′-GAACCWGCGGARGGATCA-3′) and ITS2-2043R (5′-GCTGCGTTCTTCATCGATGC-3′) primers were used to amplify the ITS1 variable region using PCR. The PCR amplification products were detected using 2% agarose gel electrophoresis, and target fragments were recovered using an AxyPrep PCR Cleanup Kit (E.Z.N.A.^®^Soil DNA Kit). The purified PCR products were quantified using a Qbit fluorescence quantitative system using the Quant-iT PicoGreen dsDNA Assay Kit. The qualified library concentration should be above 2 nM. The qualified sequencing libraries (index sequence non-repeatable) were gradient diluted, mixed in proportion to the required sequencing volume, and denatured into single strands using NaOH for computer sequencing. The MiSeq sequencer was used for 2 × 2,500 bp double-ended sequencing with the MiSeq Reagent Kit.

Using FLASH (v1.2.8) software, according to the overlap of double-ended sequences, the sequence was merged into the growing tag, and the barcode and primer sequences introduced during the library creation were removed. Vsearch (v2.3.4) was then used to filter the chimera. Vsearch uses the preprocessed clean data to define data with a sequence similarity greater than 97% as an operational taxonomic unit (OTU), and then selects the best centroid (located in the geometric center) sequence as the representative sequence of this OTU. Alpha and beta diversity were analyzed using QIIME (v1.8.0). Sequence alignment was performed using BLAST, and each representative sequence was annotated with the species from the OTU representative sequence to the RDP (ribosome database) and the NCBI-16S database.

### 2.5. Data analysis

Qiime software was used to analyze the alpha diversity index and UniFrac distance and build sample UPGMA (Unweighted Pair Group Method with Arithmetic Mean) clustering trees. LEfSe (Linear discriminant analysis Effect Size) software was used to search for biomarkers with statistical differences between the groups. R software was used to analyze the beta diversity index differences between groups. Tukey’s test and the R software package agricolae Wilcox, were used to perform t test analysis and mapping between the groups. R software was used to draw the relative abundance histograms and heat maps, OTU petal figures, alpha diversity curves and the Non-metric MultiDimensional Scaling (NMDS) figures. The top 100 microbial genera were selected to calculate the correlation coefficient, and then the connection points with a correlation coefficient greater than 0.6 or less than −0.6 and statistical significance (*p* < 0.05) were selected to draw the network map using graphviz-2.38.0. The prediction analysis of bacterial gene function was carried out using PICRUSt software, and the fungal ecological function of the existing species in the environment was queried in the literature by comparing with the FunGuild database.

### 2.6. Statistical analysis

All genomic analyses were performed following the manufacturer’s instructions. All trials were performed in triplicate, and data were analyzed using analysis of variance and Duncan’s multiple range test.

## 3. Results

### 3.1. Relative abundances of microbes in birch secondary forest due to different types of interference

In general, the dominant bacterial species in the sample plots of the different types of disturbance were similar; however, the abundance of dominant bacteria varied greatly. Among them, the relative average abundance of Acidobacteria and Proteobacteria were significantly higher than that of undisturbed sample plots, except for modified sample artificial larch forest belt (ML) sample plots, especially those disturbed by fire. The relative average abundance of Verrucobacteria in all the disturbed sample plot was significantly lower than that in the undisturbed sample plots. The relative average abundance of actinomycetes in all sample plots except ML was significantly lower than that in undisturbed sample plots.

**Figure 1:**
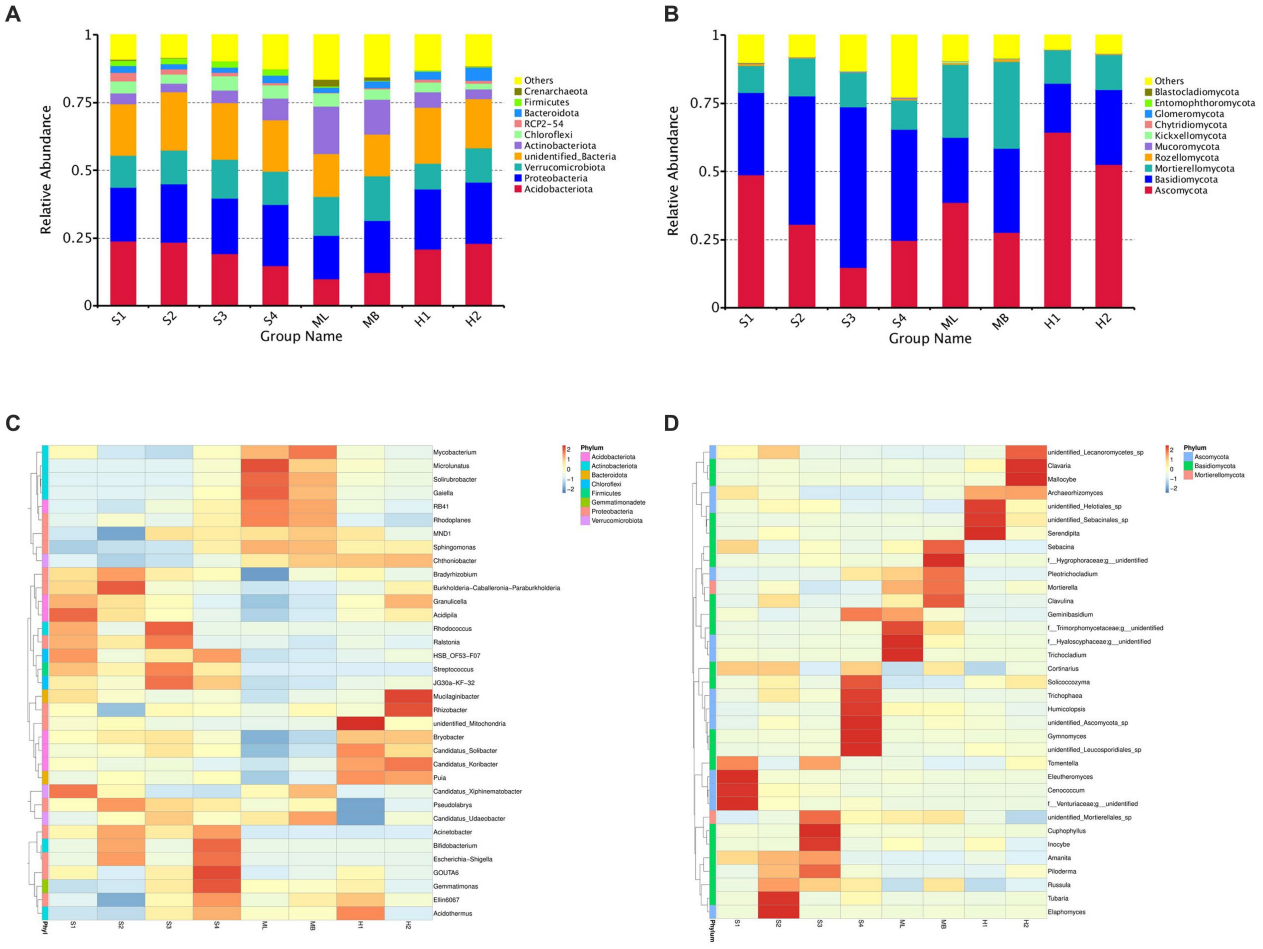
Total bacterial and fungal abundance in birch secondary forest due to different types of interference of in the Greater Khingan Mountains in China. **(A)** Relative abundance of bacteria (16S rRNA gene copy numbers) at the family taxonomic level. **(B)** Relative abundance of fungi (ITS copy numbers) at the family taxonomic level. Relative abundance of bacteria **(C,D)** fungi in birch secondary forest due to different types of interference. Names of bacteria and fungi are shown in different colors, others represent the sum of the relative abundances of all bacteria or fungi other than those of shown in the diagram. The species annotation of bacteria **(C)** and fungi **(D)** are shown at the genus taxonomic level. The tree on the left is the species cluster tree; the value corresponding to the heat map is the *Z*-value obtained from the relative abundance of species in each row after standardized processing.

The dominant bacteria in various soils at the phylum level were Acidobacteria, Proteobacteria, Verrucomicrobia, and Actinobacteriota (Figure 1A). A small number of Chioroflexi, RPC2-54, Bacteroidota, Firmicutes, and Crenarchaeota were also found. The dominant species of fungi were similar in the different disturbance sample plots, but the abundance of dominant fungi varied greatly (Figure 1B). The relative average abundance of Ascomycota in all of the disturbed sample plots, except the gradual cutting and replenishing sample plots S3 and S4, was significantly higher than that in the undisturbed sample plots, especially in the fire-disturbed sample plots. The relative average abundance of basidiomycetes was significantly lower than that of undisturbed sample plots except the gradual cutting samples S1 and S2, S3 and S4. In terms of the relative average abundance of sporidium, all disturbed sample plots were significantly lower than that of undisturbed sample plots. The dominant fungi in all soils were Ascomycota, Basidiomycota and Mortierellaomycota. There was also a small distribution of Rozellomycota, Mucoromycota, Kickxellomycota, Chytridiomycota, Glomeromycota, Entomophthoromycota, and Blastocladiomycota.

To investigate which species were richer or less concentrated in the samples, the top 35 genera with the highest abundance were selected based on the species annotations and abundance information of all samples at the genus level.

Clustering was conducted from the two levels of species and samples and are shown as heat maps (Figures 1C,D).

At the species level of bacterial abundance, we found that the abundance of *Acidipila* in S1 was significantly higher than other sample plots. The abundance of MND1 and Ellin6067 in S2 were significantly lower than other sample plots. In S3, the abundance of *Rhodococcus* and in S4, the abundance of GOUTA6 and *Gemmatimonas* were significantly higher than other sample plots. In the heavy burn sample plots H1, unidentified-mitochondria showed high abundance and *Pseudolabrys* and *Candidatus*-Udaeobacters showed lower abundance. In the moderate fire sample plot H2, the abundance of *Mucilaginibacter* and *Rhizobacter* were significantly higher than other sample plots. In ML, the abundance of *Microlunatus* showed high abundance and *Bradyrhizobium* showed low abundance.

At the fungal level of species, the abundance of *Eleutheromyces*, *Cenococcum* and *Venturiaceae* in S1, *Tubaria* and *Elaphomyces* in S2, and *Cuphophyllu* and *Inocybe* in S3 were higher than the other sample plots. In S4, the abundance of *Solicoccozyma*, *Trichophaea*, *Humicolopsis*, *Gymnomyces*, unidentified *Leucosporidiales* sp. and *Ascomycota* sp. were higher than other sample plots. In ML, the abundance of f_Trimorphomycetaceae;g_unidentified, *Trichocladium*, and f_Hyaloscyphaceae;g_unidentified, and in MB, the abundance of f_Hygrophoraceae;g_unidentified) were higher than other sample plots. In H1 and H2, the abundance of unidentified_*Helotiales_*sp., unidentified_*Sebacinales_*sp., *Serendipita*, *Clavaria*, and *Mallocybe* were higher than other sample plots.

**Figure 2:**
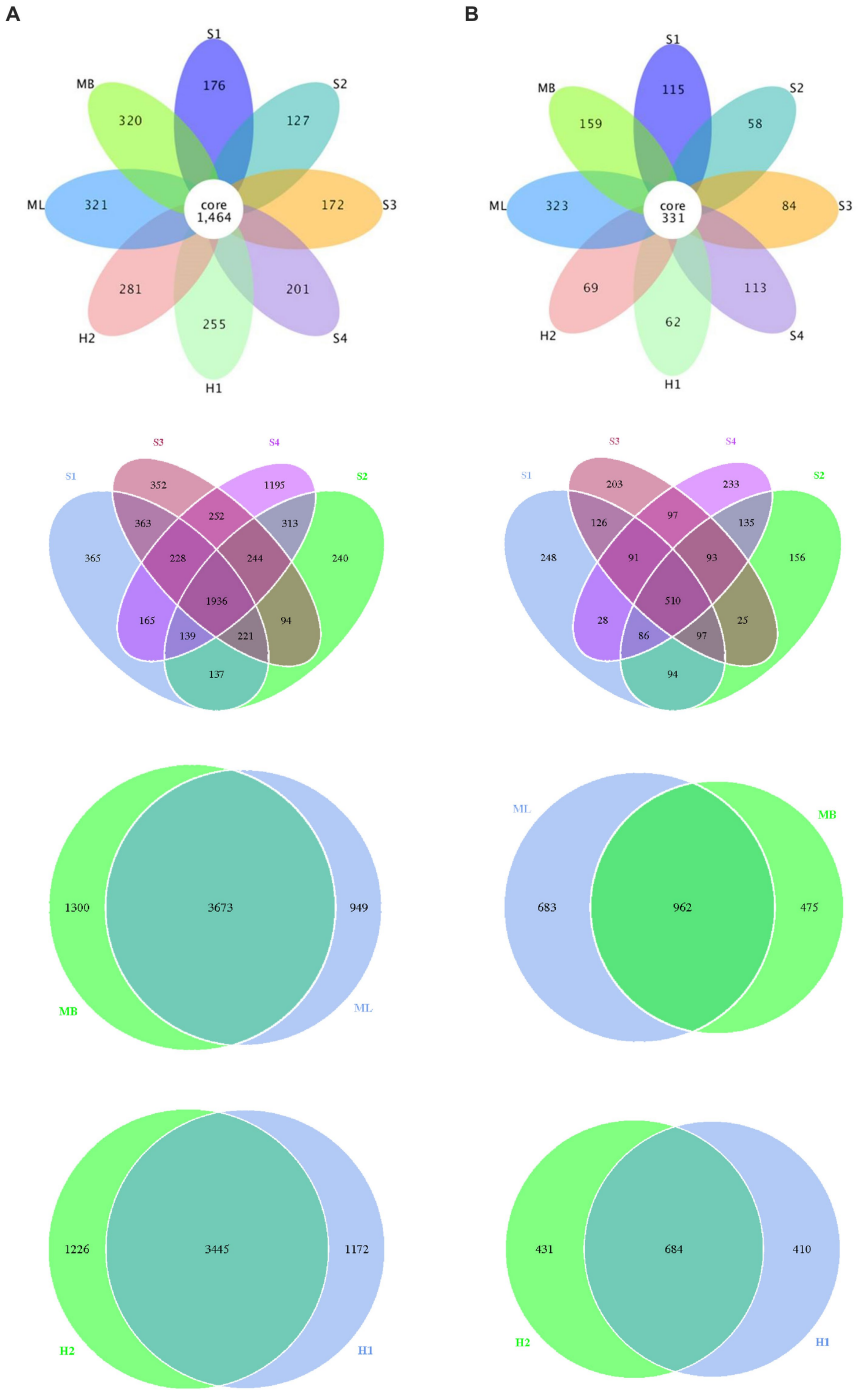
Flower petals of bacterial and fungal abundance in birch secondary forest due to different types of interference in the Greater Khingan Mountains in China. **(A)** Bacterial and **(B)** fungal petal diagrams. Each petal represents a sample (group), different colors represent different samples (groups), the core number in the middle represents the number of operational taxonomic units (OTU) shared by all samples, and the number on the petal represents the number of OTU unique to that sample (group).

We also identified different or common OTUs in the tested sample plots as shown in Figure 2. The results showed that the total number of bacterial OTUs common to the different interference type sample plots was 1,464, and individual to the separate sample plots there were 176 in S1, 127 in S2, 172 in S3, 201 in S4, 255 in H1, 281 in H2, 321 in ML, 320 in MB. There were 137 bacterial OTUs in S1 and S2, 252 in S3 and S4, 3,673 in ML and MB, 3445 in H1 and H2, respectively. There was a total of 331 fungal OTUs common to the different interference type sample plots, and individual to the separate sample plots there were 115 in S1, 58 in S2, 84 in S3, 113 in S4, 62 in H1, 69 in H2, 323 in ML, and 159 in MB. There were 94 fungal OTUs in S1 and S2, 97 in S3 and S4, 962 in ML and MB, 684 in H1 and H2, respectively.

### 3.2. Microbial diversity and community composition in birch secondary forest due to different types of interference

The number of bacterial OTUs reached a high of 3,614 and a low of 1847 (Table 2). These results indicated that the abundance of bacteria in the soil was very high, and there were significant differences in the abundance of bacteria among the different sample plots (*p* < 0.05). The larger the Shannon index, the higher the diversity of the community and the more uniform the distribution of species. Contrary to this, the larger the Simpson index, the more undiversified the community. Of the eight sample plots, the Shannon index of ML was the highest, indicating that the bacterial community diversity of the modified *Betula platyphylla Suk* secondary forest was the highest and higher than MB, while that of the other sample plots were significantly lower than MB (*p* < 0.05). The Simpson index of H1 was the highest, indicating that the bacterial community diversity of birch secondary forest after severe burning was the lowest, and there was no significant difference in other plots. The Chao1 index is used to estimate the number of OTUs contained in a community sample, which can reflect the presence of low-abundance species in the community. Similar to the Chao1 index, the ACE index reflects the overall situation of the community. The Chao1 and ACE indexes were highest in MB with the remaining sample plots significantly lower (p < 0.05).

**Table 2:** Alpha diversity index of bacteria.

Sample plot	OTU	Shannon index	Simpson index	Chao1 index	ACE index
S1	2224.333 ± 227.300 cd	8.341 ± 0.695 cd	0.987 ± 0.010a	2430.247 ± 245.040c	2469.482 ± 253.009c
S2	2080.000 ± 206.007d	8.168 ± 0.153d	0.988 ± 0.001a	2284.634 ± 243.571c	2320.714 ± 247.204c
S3	2317.667 ± 82.500 cd	8.520 ± 0.138bcd	0.991 ± 0.001a	2546.394 ± 88.585c	2588.771 ± 98.700c
S4	2637.000 ± 490.739bc	9.025 ± 0.460abc	0.994 ± 0.002a	2899.780 ± 544.695bc	2933.270 ± 565.524bc
ML	3120.000 ± 28.618ab	9.361 ± 0.210a	0.993 ± 0.002a	3420.776 ± 64.859ab	3460.539 ± 51.166ab
MB	3270.667 ± 303.133a	9.329 ± 0.251a	0.992 ± 0.004a	3713.612 ± 486.754a	3764.657 ± 513.098a
H1	3066.667 ± 70.494ab	9.211 ± 0.265ab	0.994 ± 0.001a	3375.281 ± 59.350ab	3422.658 ± 57.061ab
H2	2940.000 ± 349.089ab	9.031 ± 0.321abc	0.993 ± 0.001a	3264.878 ± 383.517ab	3322.197 ± 374.370ab

The data in the table are mean ± standard deviation; Different letters in the same column indicate significant difference (*p* < 0.05). OTU, operational taxonomic unit.

The number of fungal OTUs reached a high of 975 and a low of 579 (Table 3). The results indicated that the abundance of fungi in the soil was high, and there were significant differences among the different sample plots (*p* < 0.05). Of the eight sample plots, the Shannon index of S3 was the lowest, indicating that the fungal community diversity of the birch secondary forest was the lowest, and all sample plots were lower than MB, except ML. The Simpson index of S1 was the highest, indicating that the fungal community diversity of birch secondary forest was the highest after gradual cutting, but there was no significant difference in the other sample plots. The Chao1 and ACE indexes were all significantly lower than MB, except ML (*p* < 0.05).

**Table 3:** Alpha diversity index of fungi.

Sample plot	OTU	Shannon index	Simpson index	Chao1 index	ACE index
S1	713.333 ± 136.258bc	5.259 ± 1.455a	0.884 ± 0.136a	781.781 ± 147.527bc	796.618 ± 143.562bc
S2	702.333 ± 84.996bc	5.488 ± 0.292a	0.931 ± 0.027a	793.372 ± 75.586bc	814.472 ± 70.949bc
S3	716.000 ± 99.232bc	4.866 ± 0.524a	0.887 ± 0.056a	812.512 ± 99.000bc	814.987 ± 108.086bc
S4	748.667 ± 49.903bc	5.574 ± 0151a	0.945 ± 0.010a	817.058 ± 59.834bc	830.622 ± 59.918bc
ML	954.000 ± 23.896a	6.210 ± 0382a	0.954 ± 0.030a	1039.604 ± 24.885a	1053.820 ± 28.545a
MB	838.00 ± 84.113ab	5.589 ± 0.440a	0.944 ± 0.020a	978.894 ± 162.274ab	991.733 ± 164.673ab
H1	630.667 ± 46.458c	5.137 ± 0.233a	0.937 ± 0.012a	708.904 ± 52.653c	737.602 ± 60.505c
H2	644.000 ± 98.853c	5.006 ± 0.888a	0.904 ± 0.063a	702.615 ± 103.692c	719.499 ± 106.940c

The data in the table are mean ± standard deviation; Different letters in the same column indicated significant difference (*p* < 0.05). OTU, operational taxonomic unit.

**Figure 3:**
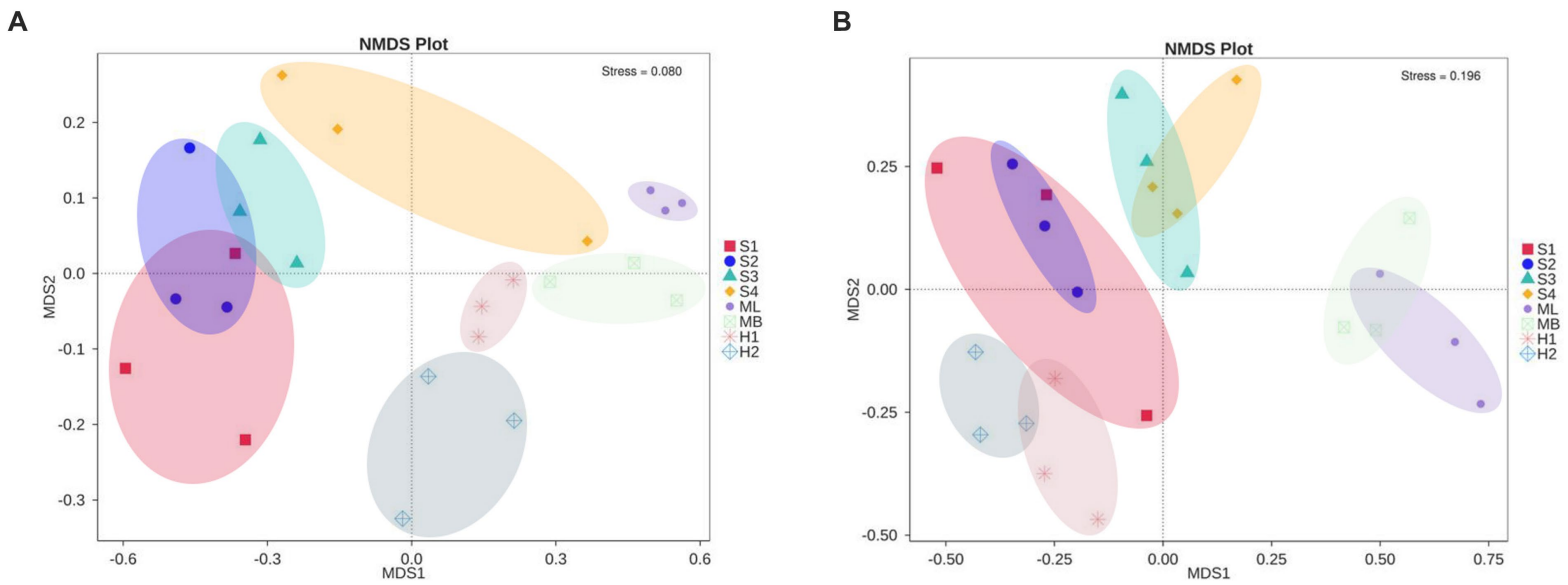
Changes in microbial community structure in birch secondary forest in due to different types of interference in the Greater Khingan Mountains in China. **(A)** Bacterial and **(B)** fungal NMDS plots. NMDS 2D stress = 0.11. Vectors show maximal correlation of the ordination configuration of variables with the strongest relationships to community structure. Taxonomic codes plotted on the ordination show the abundance-weight centroids of enriched taxa. NMDS, Non-metric MultiDimensional Scaling.

NMDS analysis revealed a clear separation of bacterial communities between the three microhabitats in the different types of interference of birch secondary forest. The stress of most samples was less than 0.2, which indicates that the NMDS accurately reflects the degree of difference between the samples. The distance between the H1 and H2 samples was relatively close, the ML and MB samples were relatively close, and S3 was far apart from the S1, S2, and S4 samples (Figure 3A). There are obvious differences in the fungal clustering among the different samples. The distances between the H1 and H2 samples were relatively close, the ML and MB samples were relatively far apart, and the S1, S2, S3, and S4 samples were relatively far apart (Figure 3B). The degree of interpretation of the differences between the groups of bacteria in different sample plots was higher, while that of fungi was lower, and the *p* values of bacteria and fungi were almost all greater than 0.05, indicating that the inter-plot test reliability was not high (Table 4).

**Table 4:** Analysis of differences between groups in PERMANOVA.

	Bacteria		Fungi	
Vs_group	R2	Pr	R2	Pr
S1-S2	0.18248 (0.81752)	0.4014	0.22992 (0.77008)	0.3000
S1-S3	0.19606 (0.80394)	0.6000	0.25857 (0.74143)	0.1000
S1-S4	0.31726 (0.68274)	0.1000	0.32918 (0.67082)	0.0014
S1-ML	0.71027 (0.28973)	0.0014	0.42133 (0.57867)	0.1000
S1-MB	0.58685 (0.41315)	0.0014	0.39795 (0.60205)	0.0014
S1-H1	0.37652 (0.62348)	0.0014	0.39529 (0.60471)	0.1000
S1-H2	0.30412 (0.69588)	0.0014	0.28097 (0.71903)	0.2000
S2-S3	0.3935 (0.6065)	0.1000	0.30961 (0.69039)	0.0014
S2-S4	0.43155 (0.56845)	0.1000	0.37613 (0.62387)	0.0014
S2-ML	0.83277 (0.16723)	0.0014	0.48016 (0.51984)	0.0014
S2-MB	0.71905 (0.28095)	0.1000	0.43988 (0.56012)	0.0014
S2-H1	0.52748 (0.47252)	0.0014	0.39673 (0.60327)	0.1000
S2-H2	0.45494 (0.54506)	0.1000	0.27187 (0.72813)	0.0014
S3-S4	0.26162 (0.73838)	0.1014	0.3029 (0.6971)	0.1000
S3-ML	0.79821 (0.20179)	0.1000	0.436 (0.564)	0.1000
S3-MB	0.65981 (0.34019)	0.0014	0.40907 (0.59093)	0.1000
S3-H1	0.44149 (0.55851)	0.1000	0.43717 (0.56283)	0.1000
S3-H2	0.43977 (0.56023)	0.1000	0.35388 (0.64612)	0.1000
S4-ML	0.56257 (0.43743)	0.1000	0.45326 (0.54674)	0.0014
S4-MB	0.38862 (0.61138)	0.1014	0.40143 (0.59857)	0.1000
S4-H1	0.28737 (0.71263)	0.1000	0.49253 (0.50747)	0.0014
S4-H2	0.37544 (0.62456)	0.1000	0.39846 (0.60154)	0.1000
ML-MB	0.21819 (0.78181)	0.1014	0.22125 (0.77875)	0.2014
ML-H1	0.73749 (0.26251)	0.0014	0.57493 (0.42507)	0.0014
ML-H2	0.76689 (0.23311)	0.0014	0.48475 (0.51525)	0.0014
MB-H1	0.55748 (0.44252)	0.0014	0.55481 (0.44519)	0.0014
MB-H2	0.60428 (0.39572)	0.0014	0.44968 (0.55032)	0.1000
H1-H2	0.27435 (0.72565)	0.1000	0.28245 (0.71755)	0.0014

R2 represents the explanation degree of different groups to sample differences, that is, the ratio of group variance to total variance. The larger R2 is, the higher the explanation degree of group differences is. Pr means *p*-value, less than 0.05 indicates that the reliability of this test is high. The value corresponding to the residual term is in parentheses.

**Figure 4:**
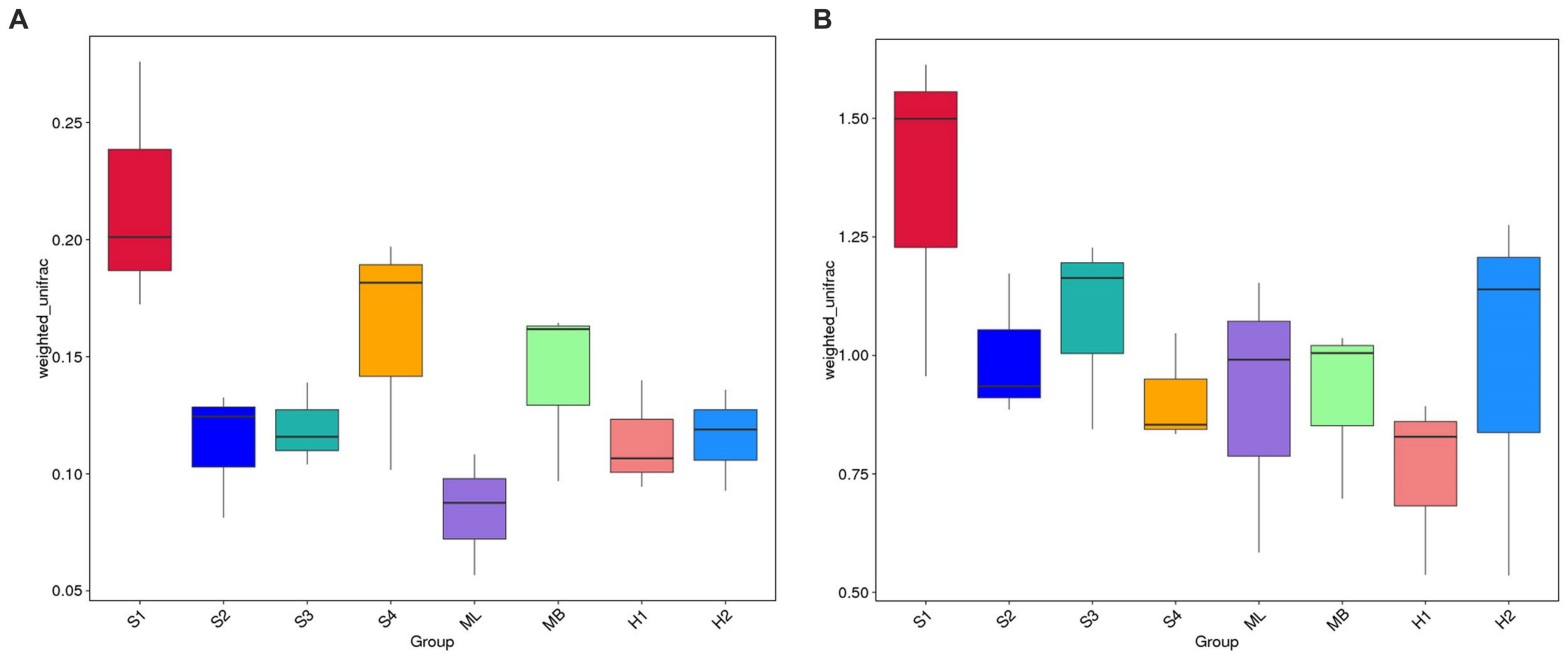
Box diagrams based on weighted UniFrac beta diversity. **(A)** Bacteria. **(B)** Fungi.

The weighted UniFrac distance matrix was used to create a box plot of beta diversity, which was used to analyze whether there were significant differences in beta diversity between the groups. As shown in Figure 4A, the box plot of sample S1 is higher on the whole, and the weighted UniFrac has a longer spatial distance, indicating that the beta diversity of the bacterial community in sample S1 is the most abundant among all samples. The height of the box plot of the ML sample was significantly lower than that of other samples, indicating that the community of the ML sample had more similar bacterial species composition, and its beta diversity was lower. Compared with S4 and MB, S2, S3, H1, and H2 have a less weighted UniFrac in spatial distance and lower beta diversity. In Figure 4B, the box plot of sample S1 was higher on the whole, indicating that the beta diversity of the fungal community in the sample was relatively rich. The height of the box plot of the H1 sample was significantly lower than that of other samples, indicating that the community of the H1 sample had more similar fungal species composition, and its beta diversity was lower. Compared with ML and H2, S2, S3, S4, and MB have reduced spatial distance and lower beta diversity.

### 3.3. Preferential microbial taxa in birch secondary forest due to different types of interference

**Figure 5:**
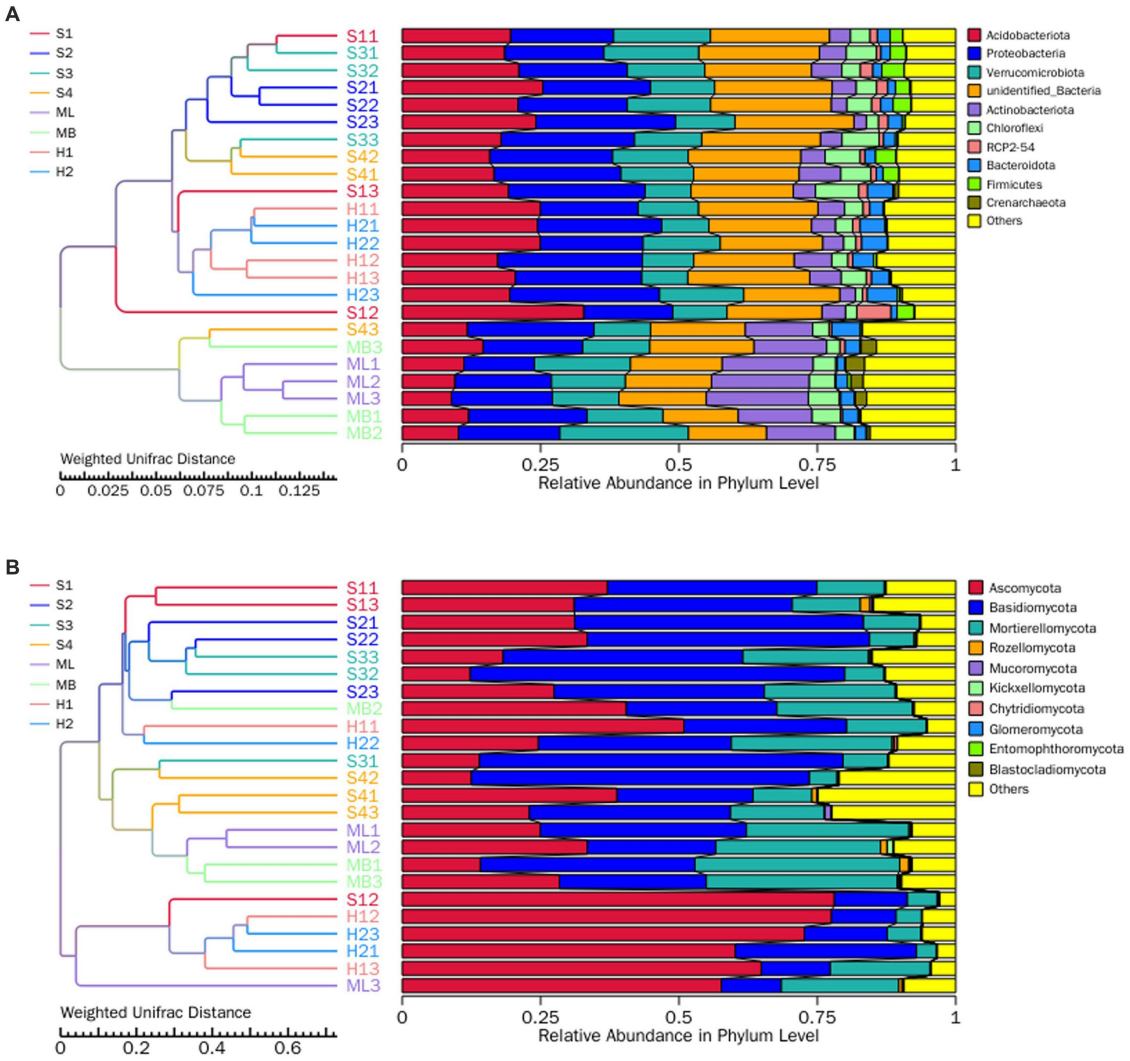
UPGMA clustering tree of the bacteria **(A)** and fungi **(B)** at the phylum level as affected by different types of interference of birch secondary forest in the Greater Khingan Mountains in China. On the left is the UPGMA cluster tree structure, and on the right is the relative abundance distribution of each sample at the phylum level. UPGMA (Unweighted Pair-group Method with Arithmetic Mean) is a commonly used clustering analysis method in environmental biology. It studies the similarity between different samples by clustering analysis of samples and constructing cluster trees of samples. The UPGMA cluster tree presented in this paper is based on Weighted Unifrac distance calculation, which is widely used in microbiome studies.

In order to study the similarity between the different groups, cluster analysis was carried out between the sample groups by constructing a cluster tree of the samples. Weighted UniFrac distance was used for clustering to take into consideration species abundance, and the clustering results were integrated with the weighted UniFrac distance relative species abundance of each sample at the phylum level. As shown in Figure 5A, when Weighted UniFrac distance was 0.029, all plots were divided into two parts, ML and MB one part, and other plots one part. When the Weighted UniFrac distance was 0.007, there were S1, S2, S3 and S4, and H1 and H2. Among them, S2 and S3, H1 and H2, ML and MB had more similar bacterial community composition and abundance, respectively. As shown in Figure 5B, when Weighted UniFrac distance was 0.102, all plots were divided into two parts, H1 and H2 one part, and other plots one part. When Weighted UniFrac distance was 0.061, one sample was S1, S2 and S3, and one was S4, ML and MB. Among them, S2 and S3, ML and MB, H1 and H2 had more similar fungal community composition and abundance, respectively.

**Figure 6:**
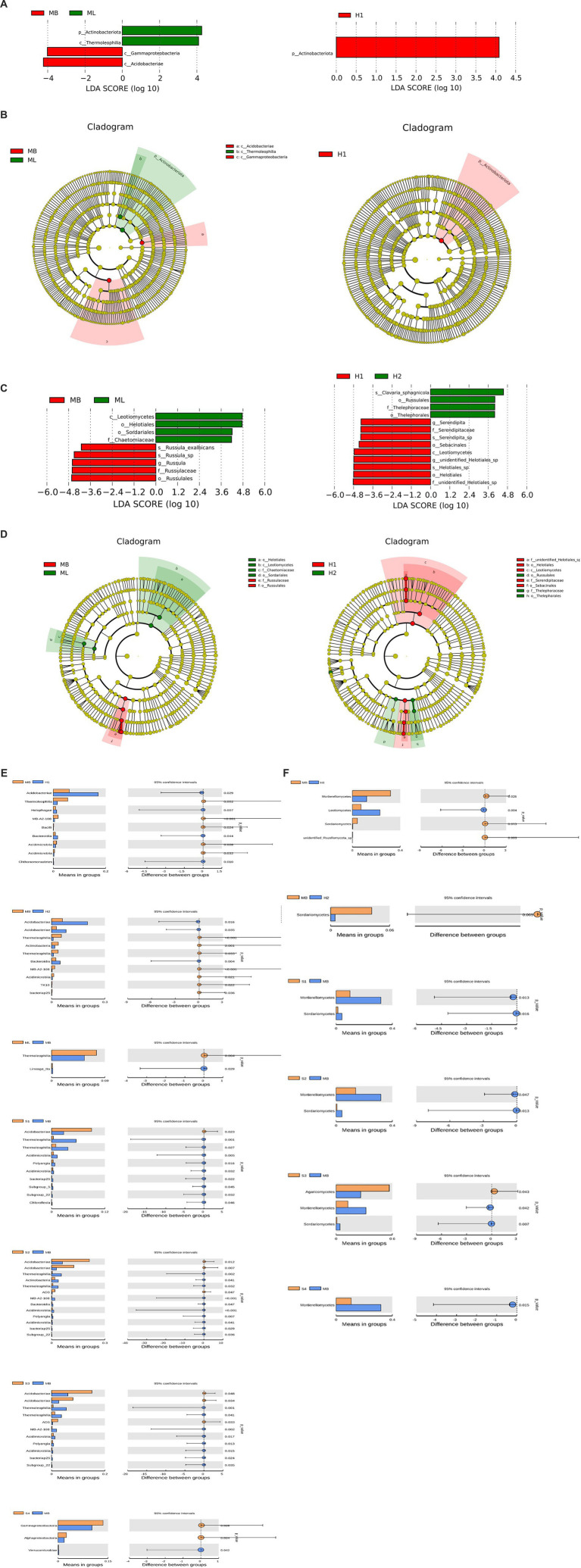
Analysis of different species between groups. **(A)** Histogram of bacterial linear discriminant analysis (LDA) value distribution. **(B)** Branching diagram of bacterial evolution. **(C)** Histogram of fungal LDA value distribution. **(D)** Branching diagram of fungal evolution. Analysis of species differences between t test groups of bacteria **(E)** and fungi **(F)**.

To analyze the different species between the groups, LEfSe was used to analyze the differences in community structure between bacteria and fungi, and species with significant abundance changes among the different groups were identified. The histogram of linear discriminant analysis (LDA) value distribution showed that there were four biomarkers with statistical difference between the MB and ML bacterial communities, and the most influential species were Actinobacteriota and Acidobacteriae. There was one biomarker with a statistical difference between the H1 bacterial communities, and the most influential species was Actinobacteriota (Figure 6A). The evolutionary clade shows that two important clades were Acidobacteriae and Thermoleophilia between the MB and ML bacterial communities. The important members of Acidobacteria include Gammaproteobacteria. An important clade between the H1 bacterial communities was Actinobacteriota (Figure 6B).

The histogram of LDA value distribution showed that there were nine biomarkers with statistical differences between the MB and ML fungal communities, and the most influential species were Leotiomycetes and Russulales. There were 13 biomarkers with statistical differences between the H1 and H2 fungal communities, and the most influential species were f-unidentified-Helotiales-sp., o-Helotiales, g-unidentified-Helotiales-sp., s-Helotiales-sp., and c-Leotiomycetes (Figure 6C). Two important clades between the MB and ML fungal communities are Leotiomycetes and Russulales. Important members of Leotietes include Helotiales, Sordariales, and Chaetomiaceae. Important members of Russulales include Russulaceae. Two important clades between the H1 and H2 fungal communities are Leotiomycetes and Russulales. Important members of Helotiales include f-unidentified-Helotiales-sp., o-Helotiales, c-Leotiomycetes, f-Serendipitaceae, and o-Sebacinales. Important members of Russulales include o-Russulales, f-Thelephoraceae and o-Thelephorales (Figure 6D).

A t test was used to identify significant species differences between the groups at the phylum level (*p* < 0.05). Among the bacteria, the Acidobacteriae abundance of H1, H2, S1, S2, and S3 was higher than that in MB (*p* < 0.05), while the Thermoleophilia and Acidimicrobiia abundance of H1, H2, S1, S2 and S3 was lower than that in MB (*p* < 0.05) (Figure 6E). Among the fungi, the abundance of Leotiomycetes in H1 was higher than MB (*p* < 0.05), and Agaricomycetes in S3 was higher than MB (*p* < 0.05). The Sordariomycetes abundance of H1, H2, S1, S2 and S3 was lower than MB (*p* < 0.05), and the Mortierellomycetes abundance of H1, S1, S2, S3 and S4 was lower than MB (*p* < 0.05) (Figure 6F).

### 3.4. Analysis of putative functional genes in birch secondary forest due to different types of interference

**Figure 7:**
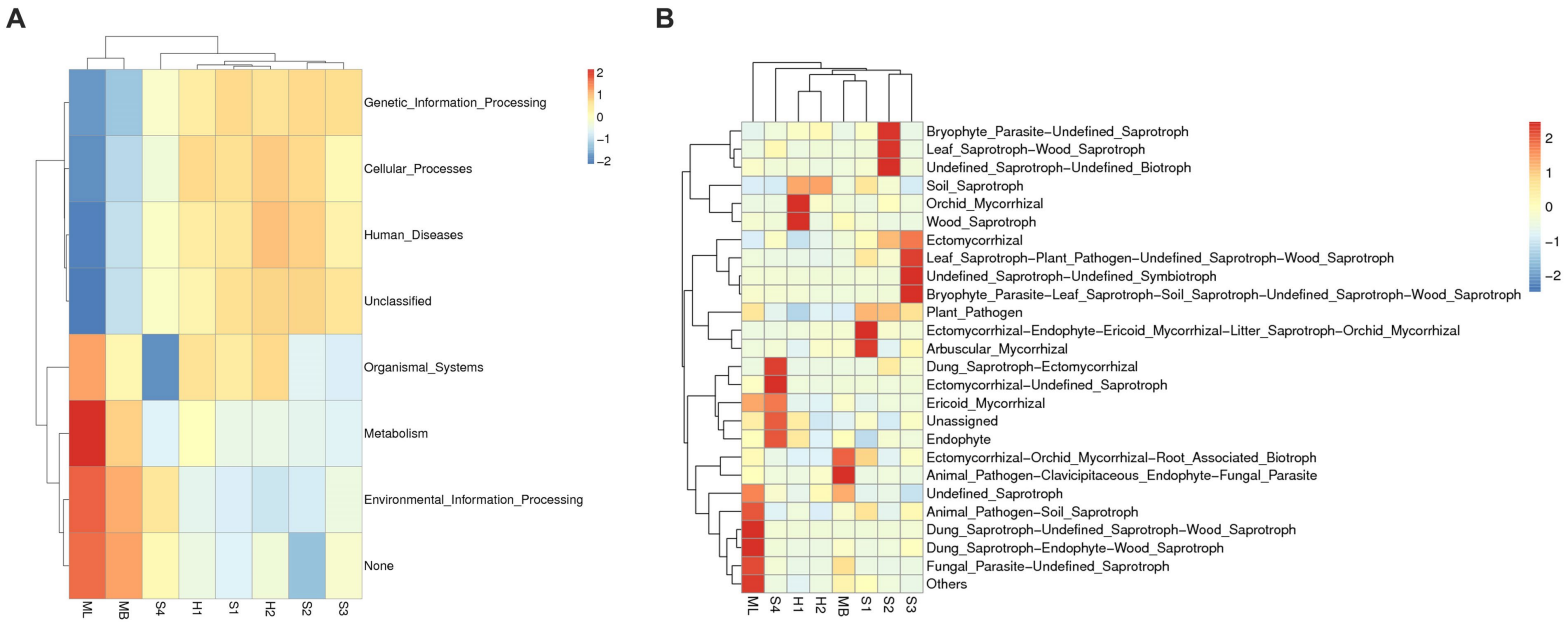
Analysis of putative functional genes. **(A)** Clustering heat maps of bacterial genes using PICRUSt function annotation. **(B)** Clustering heat maps of fungal genes using FunGuild annotation.

The PICRUSt method was used to predict the gene functions of the soil bacterial communities of *Betula platyphylla* secondary forest under the different types of disturbance. Figure 7A shows that the functional abundance results at level1 include six main categories of functions (Unclassified and None are not included): Genetic Information Processing, Cellular Processes, Human Diseases, Organismal Systems, Environmental Information Processing, and Metabolism. The results of the PICRUSt functional annotation cluster heat map show that there were some differences in bacterial community gene function among the different sample plots, among which the ML sample plot bacteria had rich functions in metabolism and environmental information processing but had monotonous functions in human diseases. The function of bacteria in the organismal systems of S4 was monotonous. The functional richness of bacteria in H1, H2, S1, S2 and S3 was similar.

The FunGuild method was used to predict the ecological functions of soil fungi in *Betula platyphylla Suk* secondary forest under different types of disturbance. The results of functional abundance in terms of Guild (functional subclass) are shown in Figure 7B. There are 23 class functions (excluding Unassigned, Undefined_Saprotroph, and Others). The FunGuild functional annotation cluster heat map shows that there were some differences in the ecological functions of fungi in the different sample plots. The ML sample plot had rich functions in fungi in animal pathogen-soil saprotroph and dung saprotroph-undefined-saprotroph-wood saprotroph. The MB sample plot was abundant in ectomycorrhizal-orchid mycorrhizal-root associated biotrophs and animal pathogen clavicipitaceous-endophyte-fungal parasites. S1 was abundant in ectomycorhizal-endophyte-ericoid mycorhizal-litter saprotroph-orchid mycorhizal and arbusular mycorhizal. S2 was abundant in bryophyte parasite-undefined saprotroph and leaf saprotroph-wood saprotroph. S3 was abundant in ectomycorrhizal, leaf saprotroph-plant pathogen-undefined saprotroph-wood saprotroph and bryophyte parasite-leaf saprotroph-soil saprotroph-undefined saprotroph-wood saprotroph. S4 was abundant in dung saprotroph-ectomycorrhizal, ectomycorrhizal-undefined saprotroph, and endophyte. H1 was abundant in orchid mycorrhizal and wood saprotroph.

### 3.5. Correlation analysis of microbes in birch secondary forest due to different types of interference

**Figure 8:**
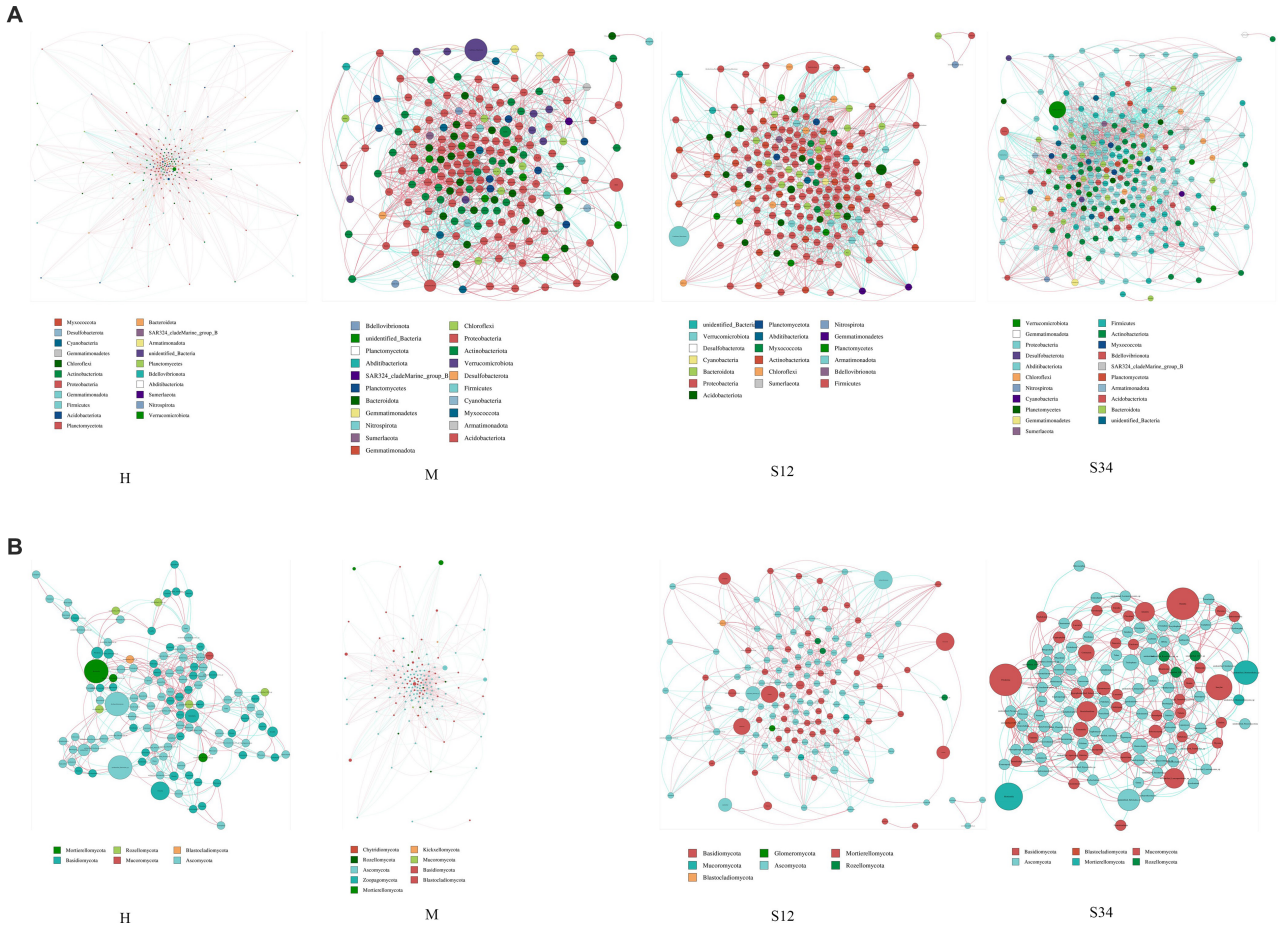
Network analysis of correlations and model prediction. **(A)** Network analysis of correlations in bacteria. **(B)** Network analysis of correlations in fungi. Nodes of the same phylum have the same color (as shown in the legend). The correlation coefficients between the thickness of the lines between nodes and the interaction between species are positively correlated in absolute value, and the color of the lines indicates the correlation (red is positively correlated, blue is negatively correlated).

Cogenetic network maps provide a new perspective to study the community structure and function of complex microbial environments. Since the co-occurrence relationship of microorganisms in different environments varies greatly, the influence of different environmental factors on microbial adaptability can be directly observed through the network diagram of species co-occurrence, as well as the dominant species and closely interacting species groups in a certain environment. These dominant species and species groups often play a unique and important role in maintaining the stability of the microbial community structure and function in the environment (Qin et al., 2012; Jiao et al., 2016). The results showed that in the microbial network, there were 16, 21, 19, and 21 bacterial phyla and 6, 9, 7, and 6 fungal phyla in sample plots H, M, S12, and S34, respectively, and there were 284 genera of bacteria in total (Figure 8A) and 199 genera of fungi (Figure 8B). The bacterial network was mainly composed of Proteobacteria, Actinobacteriota, Firmicutes, Bacteroidota, Planctomycetes, and Acidobacteriota at the phylum level. At the genus level, it was mainly composed of *Candidatus*_Udaeobacter, *Bradyrhizobium*, *Bryobacter*, *Candidatus*_Solibacter, Ellin6067, *Gemmatimonas*, and RB41. The fungal network was mainly composed of Ascomycota and Basidiomycota at the phylum level. At the genus level, it was mainly composed of *Archaeorhizomyces*, unidentified_*Helotiales_sp., Mortierella*, *Piloderma*, *Russula*, *Inocybe*, unidentified_*Mortierellales_sp*, and *Cortinarius*.

The important topological properties were calculated to describe the complex interrelation patterns between nodes. Table 5 shows that the bacterial network diameter of sample plot M was the largest, with 17 edges, while sample plot S34 was the smallest, with 13 edges. The fungal network diameter of sample plot M was the largest with 19 edges, while sample plot S34 was the smallest with 16 edges. The bacterial mean path length of plot S12 was the largest with 5.052503 edges, and the smallest was in plot S34 with 4.584009 edges. The fungal mean path length of sample plot M was the largest with 6.06031 edges, and the smallest was in sample plot S12 with 4.854684 edges. The clustering coefficient of bacteria in sample plot S34 was the largest (0.5282503), and the smallest (0.4888567) in sample plot M. The clustering coefficient of fungi in sample plot H was the largest (0.48631), and that in sample plot S34 was the smallest (0.454237). The modularity of bacteria in sample plot S12 was the largest (0.5636978), and sample plot M the smallest (0.5020276). The modularity of fungi in sample plot H was the largest (0.6185923), and that of plot M the smallest (0.53398) (a value >0.4 indicates that the network has a modular structure). The average connectivity of bacteria in sample plot S34 was the highest (21.90681), and that in sample plot H was the lowest (17.10569). The average connectivity of fungi in plot M was the largest (13.07772), while that in plot S34 was the smallest (11.05208).

**Table 5:** Data of network analysis.

		Network diameter (ND)	Modularity (MD)	Clustering coefficient (CC)	Network graph density (GD)	Average connectivity (AD)	Mean path length (APL)
Bacteria	M	17	0.5020276	0.4888567	0.03530648	17.86508	4.926815
	H	14	0.5208587	0.4898766	0.0346269	17.10569	4.756906
	S12	15	0.5636978	0.5091265	0.03296922	17.53962	5.052503
	S34	13	0.5045832	0.5282503	0.0391193	21.90681	4.584009
Fungus	M	19	0.53398	0.4816378	0.03370546	13.07772	6.06031
	H	18	0.6185923	0.48631	0.02916511	11.31606	5.816156
	S12	18	0.5866139	0.4812926	0.03053375	11.96923	4.854684
	S34	16	0.583733	0.454237	0.02863234	11.05208	5.493769

## 4. Discussion

After different types of disturbances, the changes in microbial species and quantity in forest soil and the changes in diversity index can be used as important biological indicators to evaluate the progress of the forest ecosystem restoration. The species, quantity, and diversity of microorganisms in the secondary birch forest restored using different interference methods were significantly different from those in the undisturbed birch forest. It has been reported that the recovery rate of the soil fungal community is lower than that of the bacterial community, and fire disturbance significantly reduces soil fungal richness, while selection and clear cutting interference had no significant effect (Yu et al., 2023). In our study, the abundance of bacteria in the soil was higher than that of fungi. This might be the reason that most of the study area is birch, and only a small part is coniferous forest. The structure of organic matter in the soil of broadleaf forests is simple, and the decomposition rate of bacteria is fast. Generally, the number of bacteria is high in broadleaf forests, while the number of fungi is high in coniferous forests (Xu et al., 1984; Zhu et al., 2000; DeGrood et al., 2005).

In this study, the relative abundance of Ascomycota and Basidiomycota were significantly different from other sample plots at the phylum level after fire interference, with Ascomycota significantly higher than other sample plots, and Basidiomycota significantly lower than other sample plots. Regarding the fungal community, although ascomycetes and basidiomycetes can decompose organic matter with complex structures, ascomycetes can improve the resistance ability of the soil ecosystem to environmental stress by utilizing various soil nutrient elements (Li et al., 2017), and thus easily cope with various environmental pressures (Egidi et al., 2019). The main function of basidiomycetes is to degrade lignin (Zhang et al., 2021). After fire, the soil environment is seriously damaged. Ascomycetes, due to their strong stress resistance, widely use various nutrient elements formed in a short time, and the number of basidiomycetes increases; however, the lignin in the forest is greatly reduced, resulting in a rapid decrease in the number of basidiomycetes.

The alpha diversity of forest soil microorganisms can be used to reflect the evenness and richness of their communities (Zhu et al., 2020), and beta diversity can represent the differences between community groups (Juan, 2016). It has been reported that both logging and fire significantly reduced the soil microbial richness, species uniformity and microbial diversity index of Masson pine forest (Long, 2013). In our study, the Simpson index of soil bacteria and fungi in birch secondary forest formed after fire and gradual cutting disturbances were significantly lower than that in undisturbed sample plots. Among all sample plots, ML and S1 had the highest bacterial and fungal alpha diversity, while S2 and HI had the lowest bacterial alpha diversity, and S3 had the lowest fungal alpha diversity. The dominant groups of soil microorganisms were different in the *Betulae alba* secondary forest formed under different types of disturbances. The diversity of soil microorganisms is positively correlated with the sustainable utilization and pressure resistance of soil.

The PICRUSt and FunGuild methods were used to predict the gene function of the soil bacterial community and the ecological function of soil fungi in *Betula platyphylla Suk* secondary forest under different types of disturbance. Using PICRUSt function prediction, the soil bacterial community of the *Pinus mongolicum* plantation in Hulunbuir Sandy land mainly involved environmental information processing, metabolic and genetic information processing, and other functions (Ding et al., 2021). It was also found that metabolism was the basic function of the soil bacterial community in Chinese fir (Tan et al., 2023). Here, we found that the gene functions of the soil bacterial community were rich in metabolism and environmental information processing, but monotonous in human diseases and organismal systems. The results also showed that the ecological functions of the soil fungal communities were mainly concentrated in animal pathogen, dung saprotroph, ectomycorhizal, leaf saprotroph, and bryophyte wood saprotroph. Some studies have demonstrated that the relative abundance of soil bacteria in most metabolic function prediction pathways in mixed forest is significantly higher than that in pure eucalyptus forest, the number of arbuscular mycorrhiza in mixed eucalyptus forest is higher than that in pure eucalyptus forest, and the number of ectomycorrhiza in mixed eucalyptus forest is higher than that in evergreen broad-leaved forest (Wang, 2022). We also found that each sample plot has its own unique ecological function.

Molecular ecological network analysis can reflect the multiple interactions among microorganisms in the ecosystem (Clauset et al., 2008; Jeroen and Peer, 2008) and identify the microorganisms that play a major role in the ecosystem (Bin and Steve, 2005; Chaffron et al., 2010). Through the microbial network, we found that Proteobacteria, Actinobacteriota, Firmicutes, Bacteroidota, Planctomycetes, Acidobacteriota, Ascomycota, and Basidiomycota at the phylum level play a major role, and *Candidatus*_Udaeobacter, *Bradyrhizobium*, *Bryobacter*, *Candidatus*_Solibacter, Ellin6067, *Gemmatimonas*, RB41, *Archaeorhizomyces*, unidentified_*Helotiales_sp., Mortierella*, *Piloderma*, *Russula*, *Inocybe*, unidentified_*Mortierellales_sp.,* and *Cortinarius* played major roles at the genus level. In addition, the modularity of molecular ecological network analysis can indicate the stability or anti-interference performance of the microbial community (Aldana and Cluzel, 2003; Tanaka et al., 2012). We found that the modularity index of bacteria was lower than that of fungi, indicating that the stability or anti-interference performance of the fungal community is higher than that of bacteria.

## 5. Conclusion

In this study, we found that the dominant species of bacteria and fungi in different disturbance sample plots were similar at the phylum level, but the species abundance of bacteria was significantly different, and the species and quantity of bacteria were more abundant than that of fungi. At the genus level, the difference in bacterial abundance was not apparent in the different sample plots, but the difference in fungal abundance was evident, and different sample plots had unique strains. Among the eight plots, ML and S1 had the largest bacterial and fungal alpha diversity, slightly higher than MB, while S2 and HI had the smallest bacterial alpha diversity, and S3 had the smallest fungal alpha diversity. S1 bacteria and fungi had the highest beta diversity, ML bacteria had the lowest beta diversity, and H1 fungi had the lowest beta diversity. After fire and clear cutting, there were significant differences in soil microorganisms among groups at the phylum level, namely Acidobacteriae, Acidimicrobiia, Mortierellomycetes, and Sordariomycetes. The gene functions of the soil bacterial community are rich in metabolism and environmental information processing. Each sample plot has its own unique ecological function. The stability or anti-interference performance of the fungi community was higher than that of bacteria.

## Data availability statement

The raw data supporting the conclusions of this article will be made available by the authors, without undue reservation.

## Author contributions

KZ: Conceptualization, Writing – original draft, Writing – review & editing. YH: Conceptualization, Data curation, Funding acquisition, Writing – original draft. JWL: Investigation, Writing – original draft. JL: Investigation, Writing – original draft. ZW: Resources, Writing – original draft. LL: Resources, Writing – original draft. MG: Software, Writing – original draft. RS: Funding acquisition, Supervision, Writing – review & editing. MZ: Supervision, Writing – review & editing.
